# Gout Is Not Just Arthritis: Abnormal Cortical Thickness and Structural Covariance Networks in Gout

**DOI:** 10.3389/fneur.2021.662497

**Published:** 2021-09-16

**Authors:** Yifan Yang, Yuqi Cheng, Xiangyu Wang, Bibhuti Upreti, Ruomei Cui, Shuang Liu, Baoci Shan, Hongjun Yu, Chunrong Luo, Jian Xu

**Affiliations:** ^1^Department of Rheumatology and Immunology, First Affiliated Hospital of Kunming Medical University, Kunming, China; ^2^Department of Psychiatry, First Affiliated Hospital of Kunming Medical University, Kunming, China; ^3^Nuclear Analysis Technology Key Laboratory, Institute of High Energy Physics, Chinese Academy of Sciences, Beijing, China; ^4^Magnetic Resonance Imaging Center, The First Hospital of Kunming, Kunming, China

**Keywords:** gout, brain, cortical thickness, structural covariance networks, graph theory

## Abstract

**Background:** Hyperuricemia is the cause of gout. The antioxidant and neuroprotective effects of uric acid seem to benefit some patients with central nervous system injury. However, changes in the brain structure have not been discovered in patients with gout.

**Object:** Clarify the changes in cortical thickness in patients with gout and the alteration of the structural covariance networks (SCNs) based on cortical thickness.

**Methods:** We collected structural MRIs of 23 male gout patients and 23 age-matched healthy controls. After calculating and comparing the difference in cortical thickness between the two groups, we constructed and analyzed the cortical thickness covariance networks of the two groups, and we investigated for any changes in SCNs of gout patients.

**Results:** Gout patients have thicker cortices in the left postcentral, left supramarginal, right medial temporal, and right medial orbitofrontal regions; and thinner cortices were found in the left insula, left superior frontal, right pericalcarine, and right precentral regions. In SCN analysis, between-group differences in global network measures showed that gout patients have a higher global efficiency. In regional network measures, more nodes in gout patients have increased centrality. In network hub analysis, we found that the transfer of the core hub area, rather than the change in number, may be the characteristic of the gout's cortical thickness covariance network.

**Conclusion:** This is the first study on changes in brain cortical thickness and SCN based on graph theory in patients with gout. The present study found that, compared with healthy controls, gout patients show regional cortical thinning or thickening, and variation in the properties of the cortical thickness covariance network also changed. These alterations may be the combined effect of disease damage and physiological compensation. More research is needed to fully understand the complex underlying mechanisms of gout brain variation.

## Introduction

Gout is the most common type of inflammatory arthritis. The cause is high blood uric acid concentration due to uric acid metabolism disorder, which causes uric acid crystal deposition around the joints to cause local inflammation. The affected joints have obvious inflammation and pain; some patients develop gouty tophi. Due to differences in research methods and populations render fluctuating epidemiological indicators worldwide, the worldwide prevalence of gout ranges between 1 and 6.8%, and incidence ranges between 0.58 and 2.89 per 1,000 person-years (1, 2).

Many facts prove that gout is more than just arthritis; given the important antioxidant properties of uric acid, hyperuricemia may have some beneficial effects while causing gout. Studies have found that patients with gout are at a lower risk of Parkinson's disease (3). Mean plasma uric acid in current major depressive disorder and/or anxiety disorder(s) were lower than in remitted cases; also symptom severity and duration were negatively correlated with uric acid (4). Similarly, patients with Alzheimer's disease have lower central nervous system uric acid levels (5). These results suggest that uric acid may have neuroprotective effects, and these findings have been verified in several experiments. Liu et al. found that traumatic brain injury patients with significantly lower serum uric acid levels have a better prognosis. Controlled cortical impact mouse serum uric acid also decreased, but the uric acid concentration in the brain tissue increased; and by increasing the plasma uric acid concentration, the brain tissue uric acid concentration can be increased, and the neurological function of the mouse can be restored (6). However, the existing research results are highly heterogeneous. As gout is related to metabolic syndrome, type 2 diabetes, cardiovascular, and strokes (7), research methods of the studies reporting neuroprotective effects of uric acid have been questioned, whereas some studies have reached contradictory conclusions (8, 9).

The cerebral cortex morphological indicators such as cortical thickness, volume, and surface area can be used to quantitatively reflect the abnormalities of brain structure and provide a neuropathological basis for related disorders. Indices of brain macrostructure reflected by MRI can provide evidence of subtle changes, thus helping researchers better understand the mechanism of disease. Magnetic resonance studies have determined abnormality of brain structure in many diseases such as systemic lupus erythematosus, multiple sclerosis, and Parkinson's and Alzheimer's diseases (10–12).

Graph theory is a branch of mathematics that is used to study interacting element within systems. The graph in graph theory is composed of a number of given nodes and edges connecting two nodes. This graph usually describes the correlation between the two, suitable for brain network research. In recent years, progressively more studies have employed graph theory to model brain organization as a network of nodes and edges in the large scale (13, 14). A brain partition template is constructed to analyze correlation between these brain areas and define brain structure and functional networks. Functional MRI (fMRI) data, structural MRI (sMRI) data, and diffusion tensor imaging (DTI) data can be used to construct a brain network matrix to reflect the functional and structural networks, namely, global attributes (such as clustering coefficient, shortest path length, local efficiency, and global efficiency), modular attributes, and local node attributes. Prior studies on brain network have identified small-world attribute in human brains (15–17), which reflects the basic concepts in human brain's information processing: functional separation and functional integration (18). In this network, the local areas adjacent to the brain are closely connected, and at the same time, any two brain areas are left with a small number of connections for rapid communication. Local information processing and the whole brain information transmission achieve a balance, while meeting the efficiency of functional classification and functional integration, reducing the cost of maintaining efficient communication (19, 20). Previous neuroimaging investigations found that the small-world architecture is disrupted in various diseases, such as Parkinson's disease (21), clinically isolated syndrome (22), and acute stroke (23).

The correlation between the morphological characteristics of different brain regions is considered to be a manifestation of the phenomenon of structural covariance, reflecting the large scale of structural network model, also called structural covariance networks (SCNs) (24). Brain SCNs reflect the impact of the specific environment created by common experience such as brain development, disease state, and social environment on the brain (25, 26). The basis of structural covariance is the functional connection and direct anatomical connection of different brain areas. The application of SCN analysis provides new ideas for exploring brain development and disease. It has been widely used in the research for Alzheimer's disease, schizophrenia, depression, bilateral cerebral palsy, and other diseases (26–29).

As mentioned above, the potential effect of hyperuricemia on the central nervous system is still inconclusive, and it provides clues for our research. Gout is a chronic disease, and the changes in brain structure caused by it are more stable than functional changes. At the same time, gout is a systemic disease, and its damage on the brain is not limited to certain brain areas, so we chose graph theory network analysis. Although the relevant surface-based morphometry and high-level graph-theoretical analysis magnetic resonance studies in patients with gout are still lacking, it is based on the existing mature MRI research methods, supporting software, and the results obtained in many other neuropsychiatric studies; we expected that patients with gout may have abnormal changes in cortical thickness. It was also expected that common and unique structural covariance patterns may exist in gout patients. This is an exploratory research. In the present study, we used an exploratory approach to measure and compare cortical thickness and its SCN in a small sample of gout patients and healthy controls (HCs) to understand the brain cortex organizational features and underlying physiopathology mechanisms of patients with gout.

## Materials and Methods

### Participants

A total of 30 male volunteers diagnosed with gout were enrolled in the outpatient and inpatient Department of Rheumatology and Immunology at the First Affiliated Hospital of Kunming Medical University from 2015 to 2018. All the patients fulfilled the 2015 American College of Rheumatology/European Alliance of Associations for Rheumatology (ACR/EULAR) gout classification criteria (30). The inclusion criteria for all the patients were as follows: (1) male; (2) age ranging from 18 to 60 years; (3) right-handedness; and (4) patients willing to participate in the study voluntarily and sign informed consent.

The exclusion criteria for all subjects were as follows: (1) patients fulfilling the ACR classification criteria for systemic lupus erythematosus, rheumatoid arthritis, systemic sclerosis, primary or secondary Sjögren's syndrome, or other connective tissue diseases; (2) patients with organic encephalopathy or neurological disorders (such as a history of traumatic brain injury, surgery, Parkinson's disease, or epilepsy) that can interfere with brain structure or diffusion imaging; (3) patients with severe active mental illness (such as severe behavioral disorders and unconsciousness); (4) patients with a history of alcoholism and drug abuse; (5) patients who have contraindications to MRI (e.g., claustrophobia and metal implants); (6) patients with severe clinical conditions that can lead to brain atrophy (such as a history of hypertension, diabetes, stroke, and renal insufficiency); and (7) patients whose conventional T1- and T2-weighted MRI scan suggesting abnormal brain structure.

After the scan, we first checked whether the data file was missing. Raw MR images of all subjects were read by a senior radiologist. Three volunteers refused the MRI examination, one volunteer showed lacunar infarction on MRI, and three volunteers had partial MRI results missing. Finally, 23 gout patients were included in the study.

Twenty-three male age-matched HCs were enrolled in this study.

This study has been approved by the ethics committee of the First Affiliated Hospital of Kunming Medical University. Before beginning the trial, the participants and their legal guardians were informed of the trial procedures in detail, and signed informed consent was obtained.

### MR Image Acquisition

All MR images were obtained by an experienced neuroradiologist using a 1.5T MRI scanner (TwinSpeed; GE Medical Systems, Milwaukee, WI, USA) with cage head coils for MRI data acquisition. Subjects were placed in a supine position and advised to be relaxed, to be motionless, to have their eyes closed during the scan. Foam support pads were used to reduce head movement. Conventional T1-weighted image (T1WI) and T2-weighted image (T2WI) plain scanning was performed first to exclude obvious structural abnormalities. The scanning parameters were as follows: axial T1WI: echo time (TE) = 8.9 ms, repetition time (TR) = 2,056.9 ms, layer thickness = 5 mm, layer spacing = 6 mm, and turning angle = 90°; T2WI: TR = 12,000 ms, TE = 88.4 ms, layer thickness = 6 mm, layer spacing = 6 mm, and turning angle = 90°. No subjects were excluded for the presence of abnormal brain structure; 3D-MRI uses 3D-T1-weighted fast phase disturbance gradient echo sequence (3D-T1-fspgr sequence), and the parameters were as follows: TR = 10.5 ms, TE = 2 ms, inversion time = 350 ms, layer thickness = 1.8 mm and no layer interval, scanning matrix = 256, turning angle = 15°, field of view = 240 mm, spatial resolution = 0.94 mm × 0.94 mm × 0.9 mm, and layer number = 172; scanning range covers the whole brain.

### Image Processing

Structural images were processed using the FreeSurfer package (version 5.3.0, https://surfer.nmr.mgh.harvard.edu/fswiki/DownloadAndInstall#Download). The technical details of the image processing program have been described in previous publications (31). Briefly, the processing includes raw date import, data format conversion, head movement correction, automated Talairach transformation, non-uniform field correction, skull dissection, brain tissue segmentation, and automated reconstruction of the pial and gray matter–white matter surfaces. Then, all data were resampled and smoothened onto the FreeSurfer average subject template. In order to perform group-level whole-brain analysis, before statistical analysis, maps were filtered using a surface-based full-width at half maximum (FWHM) Gaussian kernel of 10 mm. The final maps were averaged across participants to align cortical folding patterns using a non-rigid high-dimensional spherical method (32). This software was proven to have good re-measurement reliability between magnetic field intensity and scan sequence parameters of magnetic resonance scanners of different manufacturers (33). All results of cortical segmentation were evaluated using the FreeSurfer Qoala-T tool (34) to detect the effects of head movement, reconstruction of critical regions, and mis-segmentation of non-brain tissues; and all fulfilled verification requirements. See Supplementary Material 1 for the segmentation results of some critical regions.

Cortical thickness calculation: In accordance with the principle of the surface-based morphometry, the FreeSurfer software package uses the grid-based surface analysis method proposed by Desikan et al. (35). In this method, the shortest distance from the white matter–gray matter surface to the gray matter–pia mater surface is defined as the cortical thickness. The calculation method is to use a known vertex on the outer surface as the origin and to measure the shortest distance from the outer surface to the inner surface and the shortest distance from the inner surface to the outer surface on the vertex. The average of the two measured values is defined as the thickness of the cortex at the vertex.

### Construction of Structural Covariance Networks

**Figure 1:**
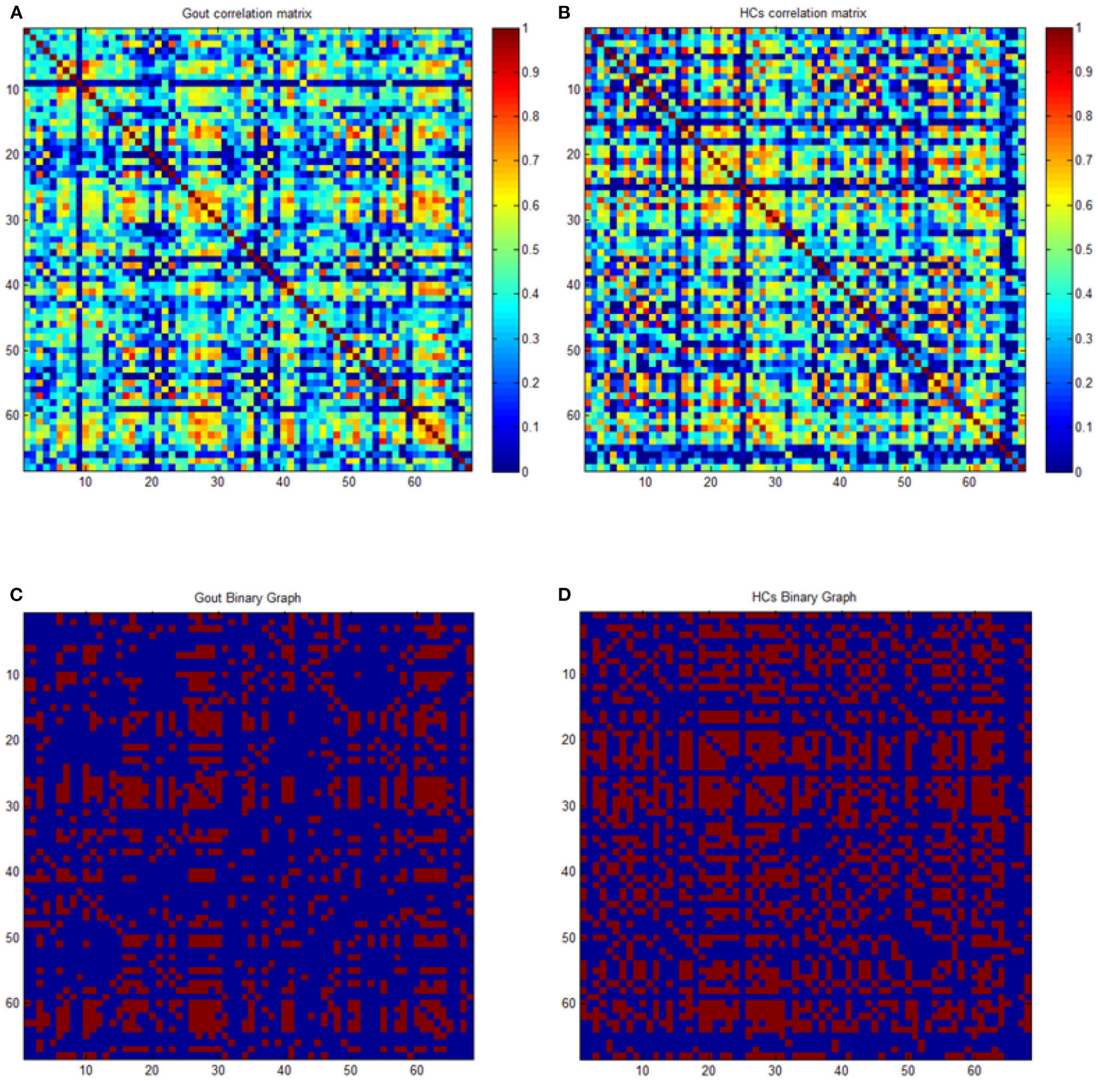
Correlation and binary matrices for gout patients and healthy controls (HCs). Correlation matrices for gout **(A)** and HCs **(B)**, and binary adjacency matrices thresholded at Dmin (0.1) for gout **(C)** and HCs **(D)**. The color bar denotes the correlation coefficient and represents the strength of the connections.

By using scripts edited based on MATLAB, the average cortical thickness of 68 brain regions covering the entire brain of each subject were extracted according to the Desikan–Killiany Atlas (35). Many studies have used 68 cortical regions of the Desikan–Killiany Atlas to construct the SCN of cortical thickness, and they demonstrated good applicability (26, 36–38). The cortical thickness data were used for construction of SCNs by Graph Analysis Toolbox (17, 39–41). By defining the 68 regions of interest as nodes, and the strength of the edges of the covariance network is defined by Pearson's correlation between the cortical thickness value of each pair of brain regions in all subjects using age as a covariate, a 68 × 68 association matrix M for each group was derived, and each entry rij is defined as Pearson's correction coefficient between the cortical thickness of regions i and j (Figures 1A,B). A minimum graph density is required for comparison of graph measures, to ensure that the two graphs being compared are fully connected (not fragmented); and for structural networks, densities above 50% might not have biological meaning. Upon referring to experience with similar studies, a threshold from 0.1 to 0.5 (with an interval of 0.02) was set as the density of the network (41, 42). Thresholding of correlation matrices with absolute values results in a dissimilarity in the number of nodes and edges, which may introduce confusion for subsequent between-group comparisons. To solve this problem, the threshold of the correlation matrix for each group can be arranged into a binary adjacency matrix with network density D, defined as the number of edges in the graph divided by the maximum possible number of edges. The network density used in this study has a wide range of 0.1 ≤ D ≤ 0.5. The lower limit of the range is defined as the minimum density at which neither of the two networks was fragmented (where Dmin = 0.1). The upper limit of the range is the maximum density of the two networks (where Dmax = 0.5). Then, threshold the association matrix into a binary matrix A with values of 1 or 0. Matrix A was derived from each association matrix where aij was considered 1 if rij was >0.1 and zero otherwise. The diagonal elements of the constructed association matrix were also set to zero (Figures 1C,D). For detailed description, see Hosseini et al. (41, 43).

From these thresholded and binarized networks, global network parameters including clustering coefficient, path length, small-world index, global efficiency, transitivity, and modularity; and regional network parameters including nodal betweenness centrality, nodal degree, and local efficiency were calculated to describe the topological characteristics of the SCN (42). Briefly, the clustering coefficient is the ratio of edges between nodes in a neighborhood divided by the number of edges that may exist between them; it represents the degree of interconnectivity between network nodes and neighboring nodes. Characteristic path length, which is the average of the shortest path between network nodes, describes the optimal path for information transmission between various nodes; the shorter the path length, the higher the efficiency of information transmission. Small-world index is the ratio of the normalized clustering coefficient to the normalized characteristic path length. The global efficiency is defined as the inverse of the harmonic mean of the shortest path length between a node and all other nodes. The local efficiency of a node is the average of the local efficiency of all nodes. The local efficiency of a global is the average global efficiency of all nodes. Transitivity is a global expression of clustering coefficient, which represents the degree of aggregation of a network as a whole. Modularity quantifies the degree to which the network can be decomposed into subnets (modules) with the maximal intra-module connections and the minimal inter-module connections. Node degree refers to the number of edges associated with a node and its interaction within the network. Nodal betweenness centrality is defined as the proportion of all the shortest paths through a given node in the network to the total number of the shortest paths (39, 44, 45).

### Statistical Analyses

Surface-based group analyses were done, using the QDEC interface provided by FreeSurfer, for differences in cortical thickness, using general linear model, controlling for age. Then, a Gaussian random field (GRF) was used at the cluster threshold of *p* < 0.01 (46) and vertex-wise threshold of *p* < 0.05 to control for multiple comparisons. Finally, Freeview was used to display the results.

To calculate significance of the differences in SCN measures between groups, we analyzed the network parameters both at Dmin and across the density range (0.1–0.5 with an interval of 0.02) using area under the curve (AUC). A non-parametric permutation test (1,000 repetitions) was used to investigate the statistical significance of the difference in global and regional network parameters. The comparison of the abovementioned parameters between groups was completed with the GAT toolbox with the result corrected by *p* < 0.05 with false discovery rate (FDR) considered to be statistically significant. In addition, the node was identified as a hub when regional node-betweenness value was at least 2^*^SD larger than the mean value.

## Result

### Demographics

We recruited 23 male patients with gout (Gout) with mean ± SD age of 42.04 ± 10.52 years. Twenty-three demographically matched male HCs with mean ± SD age of 36.96 ± 6.23 years were included. There was no statistically significant difference in age between the two groups as evidenced by the two-sample *t*-test (*p* = 0.052).

### Between-Group Comparison of Cortical Thickness

**Figure 2:**
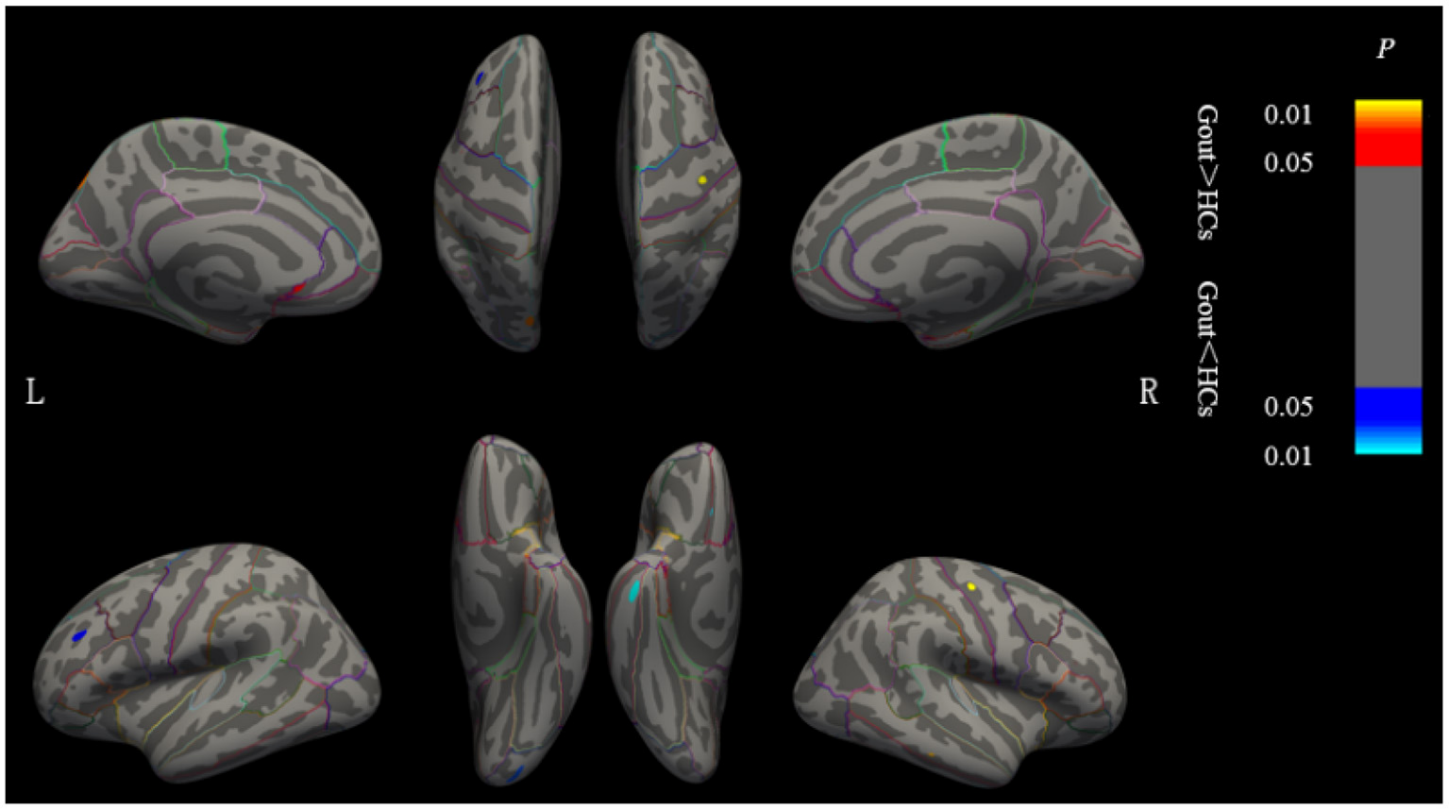
Brain regions where the cortical thickness are different between gout patients and HCs. Cluster of areas with significantly thicker cortex in gout patients, including the left postcentral, left supramarginal, right medial temporal, and right medial orbitofrontal regions. Cluster of areas with significantly thinner cortex in gout patients including the left insula, left superior frontal, right pericalcarine, and right precentral regions (GRF correction, voxel level *p* < 0.05, cluster level *p* < 0.01). Cluster with *p* < 0.01 are shown in the figure. CT, cortical thickness; HCs, healthy controls; L, left; R, right; GRF, Gaussian random field.

Clusters with significant cortical thickness differences were projected on the brain surface template (Figure 2, Table 1). Compared with the HCs, patients with gout showed a significantly thicker cortices in the left postcentral, left supramarginal, right medial temporal, and right medial orbitofrontal regions and significantly thinner cortices in the left insula, left superior frontal, right pericalcarine, and right precentral regions (GRF correction, voxel level *p* = 0.05, cluster level *p* = 0.01).

**Table 1:** Regional changes in cortical thickness in the gout group compared with HCs.

**Contrast**		**Cortical region**	**Size (mm^**2**^)**	**xyz peak (MNI)**	***p*** **-Value**
Gout > HCs	Left	Postcentral	1.40	−35.5/−30.2/54.2	0.015*
		Supramarginal	13.19	−47.9/−53.2/−32.2	0.046*
	Right	Medial temporal	6.87	59.0/−28.8/−16.5	0.025*
		Medial orbitofrontal	9.85	12.6/26.6/−16.2	0.042*
Gout < HCs	Left	Insula	2.81	−35.4/−10.4/−2.0	0.017*
		Superior frontal	10.46	−17.0/41.1/36.8	0.029*
	Right	Pericalcarine	4.98	11.5/−85.0/9.1	0.020*
		Precentral	5.29	21.4/−26.4/52.3	0.031*

*HCs, healthy controls; MNI, Montreal Neurological Institute*.

*Based on the Desikan–Killiany Atlas with 68 parcels bilaterally*.

### Between-Group Differences in Global Network Measures

**Figure 3:**
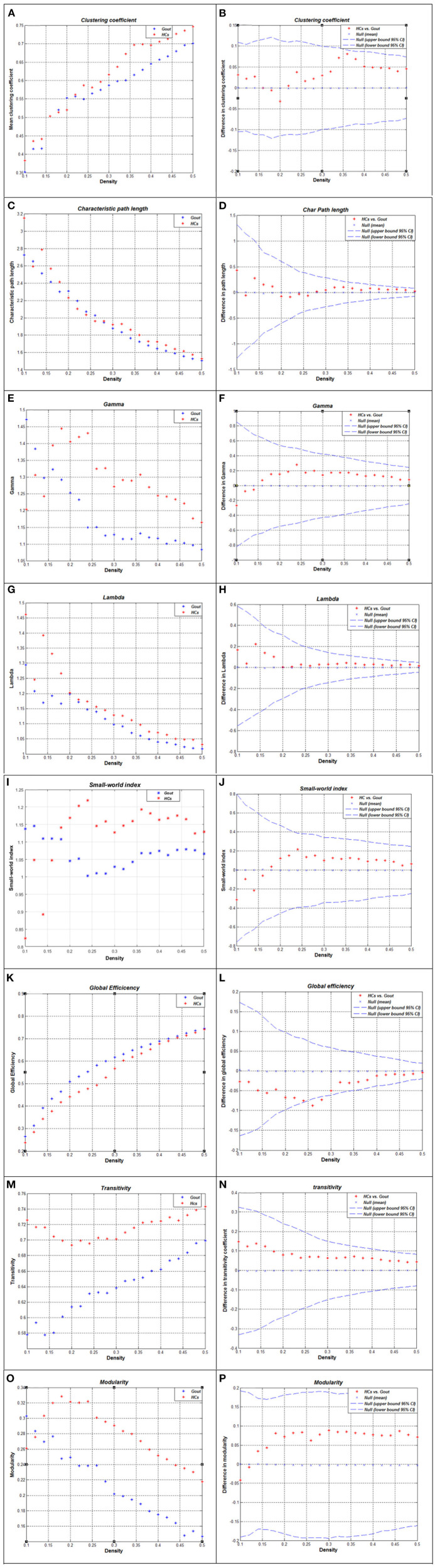
Global network measures of two groups and between-group differences in these measures. Clustering coefficient **(A,B)**, characteristic path length **(C,D)**, gamma **(E,F)**, lambda **(G,H)**, small-world index **(I,J)**, global efficiency **(K,L)**, transitivity **(M,N)**, and modularity **(O,P)** of the Gout and HC networks. The red * lying outside the confidence intervals indicates that the difference between the two groups in this density is significant **(B,D,F,H,G,L,N,P)**. Except for a few densities, the global efficiency of HCs is lower than that of the gout group (*p* < 0.05, FDR-corrected; **L**); the rest of the abovementioned measures were not significantly different between the two groups. Gout, gout group; HCs, healthy controls; FDR, false discovery rate.

The correlation matrix of the two groups shows significant correlation between most of the homotopic brain regions (Figures 1A,B). The global network measures for two groups at this range are displayed in Figure 3. Also, we compared the differences in characteristic path length, clustering coefficient, gamma (normalized clustering coefficient), lambda (normalized path length), small-world index, global efficiency, transitivity, and modularity of the networks in gout patients and HCs (Figure 3). Except for a few densities, the global efficiency in HCs was found to be lower than that in the gout group (*p* < 0.05, FDR-corrected; Figure 3L); the rest of the abovementioned measures were not significantly different between the two groups (Figure 3). AUC (density range of 0.1–0.5 with interval of 0.02) analysis also showed that there were no significant differences in all the above mentioned measures between the two groups.

### Between-Group Differences in Regional Network Measures

**Figure 4:**
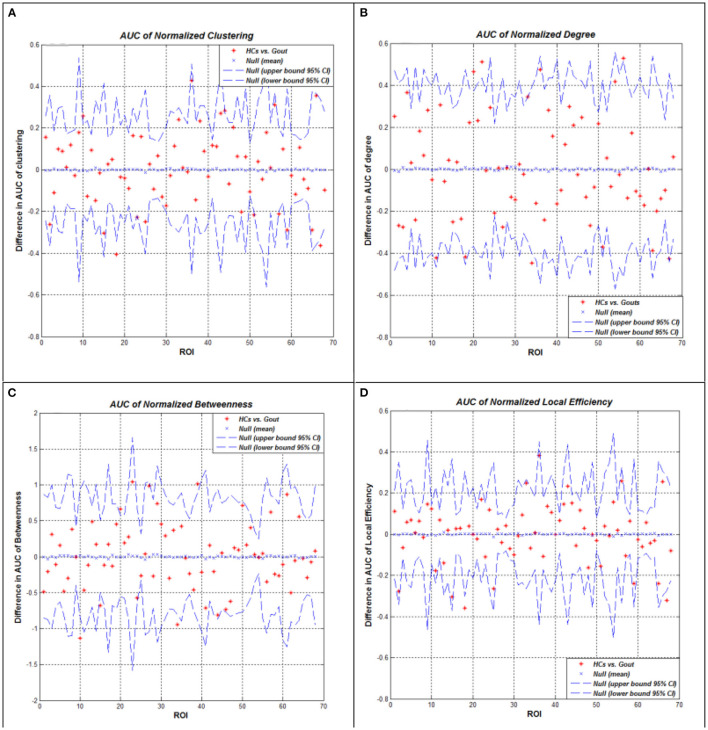
Between-group differences of measures across a range of network densities: normalized clustering coefficient **(A)**, degree **(B)**, betweenness **(C)**, and local efficiency **(D)**. The red * lying outside of the confidence intervals indicates regions in which the difference between the two groups in this density is significant. All regions survived following FDR correction (*p* < 0.05). Gout, gout group; HCs, healthy controls; AUC, area under the curve; FDR, false discovery rate.

We performed AUC (density range of 0.1–0.5 with an interval of 0.02) analysis on regional network measures. The AUC of normalized clustering coefficient in the left lateral occipital and right lateral occipital cortex regions was significantly smaller in the gout group; while in the left pars orbitalis, right pars opercularis, right precentral, and right transverse temporal cortex regions, it was significantly greater in the gout group. The AUC of normalized degree centrality in the bilateral posterior cingulate, left pericalcarine, and left insula cortex regions was significantly smaller in the gout group; while in the left transverse temporal cortex, it was significantly greater in the gout group. The AUC of normalized betweenness in the left parahippocampal and right entorhinal cortex regions was significantly smaller in the gout group, while in the left lateral occipital and left insula cortex regions, it was significantly greater in the gout group. The AUC of normalized local efficiency was not significantly smaller in any of the study region in gout patients; whereas in the left pars orbitalis, right pars opercularis, and right transverse temporal areas, it was significantly greater in the gout group (Figure 4, Table 2).

**Table 2:** Between-group differences in regional network measures.

	**Gout < HCs**	**Gout > HCs**	***p*** **-Value**
Clustering coefficient	Bilateral lateral occipital	l pars orbitalis r pars opercularis r precentral r transverse temporal	0.05
Degree	Bilateral posterior cingulate l pericalcarine l insula	l transverse temporal	0.05
Betweenness	l parahippocampal r entorhinal	l lateral occipital l insula	0.05
Local efficiency	–	l pars orbitalis r pars opercularis r transverse temporal	0.05

*Gout, gout group; HCs, healthy controls; l, left; r, right*.

### Network Hubs

**Figure 5:**
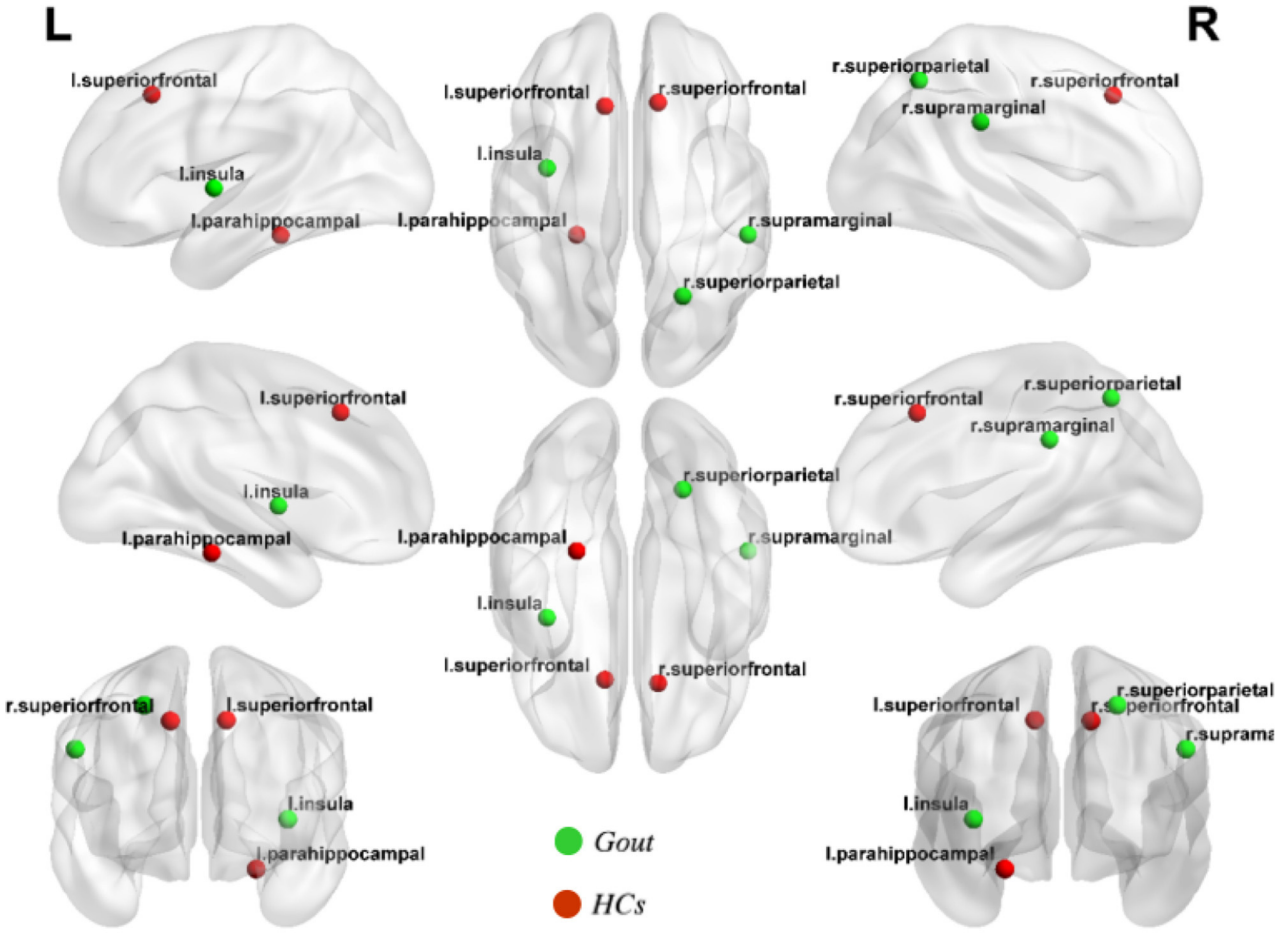
Network hubs of the gout group and healthy control (HC) group. Three network hubs were identified in each of the gout group (green highlights) and the HC group (red highlights).

Hubs are defined as regions possessing a node-betweenness 2^*^SD greater than the mean network node-betweenness. By quantifying the network hubs under the threshold of Dmin, network hubs are grouped such that each of the two groups possesses equal number of hubs, but hub distribution is unique to each group. And accordingly, the gout group hubs of node-betweenness include the left parahippocampal, left insula, and right superior parietal regions; while the health control hubs of node-betweenness include the left superior frontal, right superior frontal, and right supramarginal regions (Figure 5).

## Discussion

This is the first study on regional cortical thickness and graph theory-based large-scale SCN parameters in gout. In the present study, we found that compared with HCs, gout patients have some changes in their cortical thickness and SCN properties. Specifically, we determined that patients with gout exhibit (1) abnormal regional cortical thickness: Patients with gout showed significantly thicker cortices in the left postcentral, left supramarginal, right medial temporal, and right medial orbitofrontal regions and significantly thinner cortices in the left insula, left superior frontal, right pericalcarine, and right precentral regions. (2) Altered global network properties include increased global efficiency, increased and/or decreased regional network measures in some brain regions, and different network hubs and degree distribution. This indicates that abnormal cortical thickness and its network connection may be the consequence of gout/hyperuricemia on the brain.

Cortical thickness analysis is one of the most important brain structure analysis techniques. Humans acquire complete cortical neurons in the first half of fetal life; cortical thickness is relatively fixed during the growth and development of the brain. Some disease processes can cause changes in cortical thickness. Alzheimer's disease and dysplasia-related frontal lobe epilepsy, for example, show thinning and thickening of the cortex, respectively (47, 48). Previous studies have found that, compared with those in other animals, higher concentrations of uric acid in humans may stimulate the cerebral cortex to obtain more brain volume and better intelligence performance (49). The antioxidant, vasculoprotective, and neuroprotective effects of uric acid have also been proven to be effective in treating some neurological diseases (6, 50, 51). However, whole brain structural mapping in gout patient has not been done before. The impact comorbidities, such as hypertension, diabetes mellitus, and chronic kidney disease (7) associated with gout on brain structure is still unclear. In this study, we found that patients with gout have thickening or thinning of cortical thickness in multiple brain regions that function in sensory processing, emotional processing, language understanding, hearing, etc. Cortical thickness changes may be a potential marker for morphological changes in the brain in patients with gout. Limited by the lack of preliminary basic research and the lack of similar research, we are still not sure whether the above findings are specific, and we look forward to future studies with larger sample sizes to confirm our results.

Cluster forming threshold (CFT) is closely related to the reliability of neuroimaging research. But FreeSurfer software does not provide users with a default cluster-defining threshold. Greve and Fischl (46) used the real data analyzed in FreeSurfer to evaluate the influence of CFT and FWHM on the false-positive rate (FPR) of the results. This research found that thickness analysis showed slightly inflated FPRs in the range of 5–10% for CFTs ≤0.01 and FWHM ≥4 mm, not nearly as bad as for fMRI at matching smoothness and CFT levels. The thickness FPRs were not strongly dependent on either applied smoothing level or CFT.

Based on the research aim of this study and the threshold selection of similar studies, we set the CFT as 0.01. We believe that this threshold is appropriate to provide valid statistical results. And there is literature to support our choice. We also expect that higher thresholds can be used in future studies with larger sample sizes to better ensure the reliability of the results.

In recent years, brain connectomics has been widely utilized to study the pathological mechanism of brain diseases. At present, there is no research suggesting that the effect of uric acid on brain tissue is regionally targeted. In order to explore the multivariate network relationship between different neuroanatomical regions in the background of gout, we further conducted a SCN analysis based on cortical thickness.

After comparing several key global network measures between-groups, only in a few densities, the global efficiency of HCs was found to be significantly lower than that of the gout group (Figure 3), showing that the global cortical thickness covariance network of gout patients has not changed significantly. This may denote that the global cortical thickness is not significantly different between gout patients and HCs. In addition, coordinated compensation by relevant regions in the network may play a role in maintaining the stability of the overall network. Similar results were also found in SCN study on generalized tonic–clonic seizure, and vertically infected HIV adolescents (42, 52). The global efficiency is defined as the inverse of the harmonic mean of the shortest path length between a node and all other nodes; it is a measure that reflects parallel information transmission and comprehensive processing capabilities (19). The reduction of this measure also appears in diseases such as mild cognitive impairment (53) and peritoneal dialysis patients (54).

For regional network measures, we calculated the following measures: clustering coefficient, degree, betweenness, and local efficiency (Figure 4). These measures reflect the centrality of nodes in the network from different perspectives. As a component of the brain network, each node has a unique centrality, and it is essential to define the specialization of node functions (55). Patients with gout showed significant decrease of clustering coefficient in the bilateral lateral occipital cortex and significant increase in the left pars orbitalis, right pars opercularis, right precentral, and right transverse temporal cortex regions (Figure 4A). The clustering coefficient reflects the degree of interconnection between network nodes and neighboring nodes; the higher the clustering coefficient, the closer the connection between the local nodes of the network. In contrast, the gout group showed a decrease in the degree of most nodes (bilateral posterior cingulate, left pericalcarine, and left insula); only in the right transverse temporal area was an increase in the degree of the node seen (Figure 4B). The degree of a node is the number of edges connecting to it. The decrease in degree reflects the decrease in the centrality and importance of the node. Two nodes (left parahippocampal and right entorhinal regions decreased in gout) were found to have increased, and other two (left lateral occipital left insula regions) showed decreased betweenness (Figure 4C). Local efficiency was not reduced in any of the nodes in the gout group, but it was increased on three nodes including the left pars orbitalis, right pars opercularis, and right transverse temporal nodes (Figure 4D). In patients with gout, the centrality of some nodes was reduced, reflecting damage in anatomical connection between these nodes and other parts of the brain. However, more nodes showed increased centrality, which may be a physiological compensation to maintain the integrity of the brain structural network.

Although identical in numbers, the two groups show obvious differences in hub positioning (Figure 5). The left parahippocampal cortex is one of the hubs in gout patients group discovered in this study. Similar results were reported in previous studies on the functional brain network of osteoarthritis, in a region that was thought to be related to the response to analgesics (56, 57). The insular cortex is considered essential for the perception, modulation, and chronification of pain (58). This study indicated that the left insula is a hub; the reason may be related to this. We did not find common hubs between gout patients and HCs; the transfer of the core hub area, rather than the change in number, may be the characteristic of the gout's cortical thickness covariance network. These findings may also be caused by individual differences between subjects.

The nodes of SCN research are determined according to the brain partition atlas selected by the research method. Even for the same batch of samples, using different brain partition atlases for calculations may also have some impact on the SCN measure results. In order to provide evidence that the key results of this study are independent of the partition scheme used, we selected Desterieux Atlas (148 regions) to conduct a reanalysis of this batch of data, and the analysis results show that the key results of the two are basically consistent. Specifically, in terms of SCN global and regional network measures, the results obtained by using the two partition atlases are basically the same, but the number and location of network hubs are quite different. The reason for the high variability of network hubs between the two analyses may be due to more accurate results caused by the inclusion of more nodes and covariant connections between nodes; and on the other hand, it may be due to the limitations caused by the small sample size. But no matter which template is used, the conclusions of this study will not be subversively affected. See Supplementary Materials 2–6 for details. There are still some limitations in our study. Although we measured the serum uric acid of some gout patients included in this study before the MRI examination, the serum uric acid concentration during the acute attack of gout does not reflect the patient's long-term real situation, and most patients cannot provide long-term serum uric acid test result. We did not study the cortical thickness changes and its covariance network properties in association with uric acid concentration, which should be evaluated in future research. For surface-based morphometry method and high-level type of graph-theoretical analysis, the sample size was relatively small, and future studies with large samples will provide further insights and better test performance. To avoid the effects of age-related brain structural changes on the results of this study, we matched the age of the gout group and HCs. Although the age difference between the two groups was not statistically significant, and age was used as a covariable in subsequent statistics, and although it did show some disparity, we did not further analyze after correcting for effects of age. Nutritional status, lifestyle, and comorbidities often affect uric acid levels. This study did not collect this information, and it could not be ruled out that these differences may have impacted the results of the study.

In conclusion, this is the first study on changes in brain cortical thickness in patients with gout and changes in cortical thickness covariance networks based on graph theory. The present study found that compared with that in HCs, the regional cortical thickening or thinning was found in gout patients; the properties of the cortical thickness covariance network also changed. We were unable to recognize any protection conferred by hyperuricemia against extensive cortical thickening and SCN optimization in patients with gout. But the alterations discerned may be the combined effect of disease damage and physiological compensation and can be regarded as neuroanatomical hallmark of brain changes in gout. More research needs to be carried out to fully understand the complex underlying mechanisms of brain damage in patients with gout.

## Data Availability Statement

The raw data supporting the conclusions of this article will be made available by the authors, without undue reservation.

## Ethics Statement

The studies involving human participants were reviewed and approved by the Institutional Review Board of Kunming Medical University. The patients/participants provided their written informed consent to participate in this study.

## Author Contributions

YY, YC, and XW were responsible for the management of the research and writing the article. BU, SL, and RC were responsible for recruiting and following up the patients. HY and CL were responsible for doing MRI. BS was responsible for the consultation of the research. JX was responsible for the whole research and article. All authors contributed to the article and approved the submitted version.

## Funding

This work was supported by grants from the National Natural Science Foundation of China (81760296, 82060259, and 81460256), Yunnan Province High-level health technical talents (leading talents) (L-2019004 and L2019011), Yunnan Province Special Project for Famous Medical Talents of the “Ten Thousand Talents Program” (YNWR-MY-2018-040 and YNWR-MY-2018-041), the Funding of Yunnan Provincial Health Science and Technology Plan (2017NS051 and 2018NS0133), the Funding of Ministry of Science and Technology of Yunnan Province (2018ZF016), Yunnan Province Clinical Research Center for Skin Immune Diseases (2019ZF012), and Yunnan Province Clinical Center for Skin Immune Diseases (ZX2019-03-02).

## Conflict of Interest

The authors declare that the research was conducted in the absence of any commercial or financial relationships that could be construed as a potential conflict of interest.

## Publisher's Note

All claims expressed in this article are solely those of the authors and do not necessarily represent those of their affiliated organizations, or those of the publisher, the editors and the reviewers. Any product that may be evaluated in this article, or claim that may be made by its manufacturer, is not guaranteed or endorsed by the publisher.
