# Supplementary material for: Gout Is Not Just Arthritis: Abnormal Cortical Thickness and Structural Covariance Networks in Gout
Source: Front Neurol. 2021 Sep 16;12:662497. doi: 10.3389/fneur.2021.662497 (PMC8481804; doi:10.3389/fneur.2021.662497)
Supplement: Supplementary file 1 [file Data_Sheet_1.doc]

| 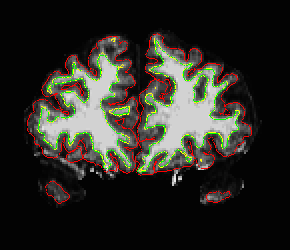 | 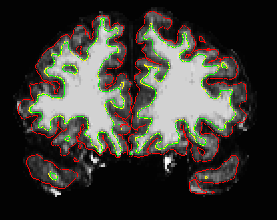 |
| --- | --- |
| 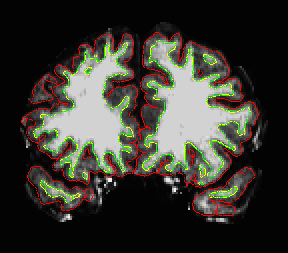 | 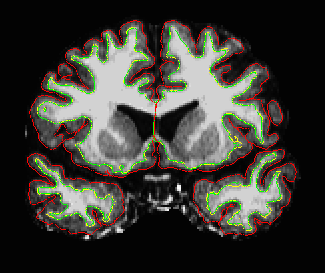 |

**Supplementary material. 1.** Cortex segmentation results, with pial surface (red) and WM/GM division (yellow). The reconstructed image is not affected by movement, Temporal pole is not missing in reconstruction, and non-brain tissue is not included in the reconstruction of the pial surface.


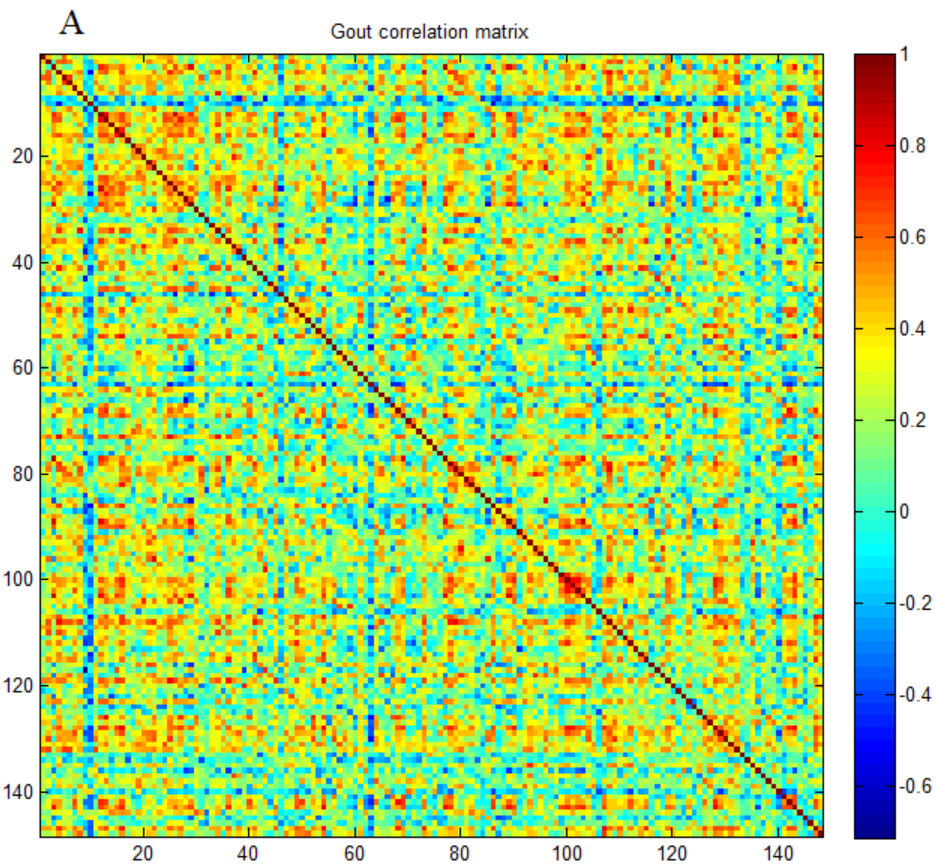

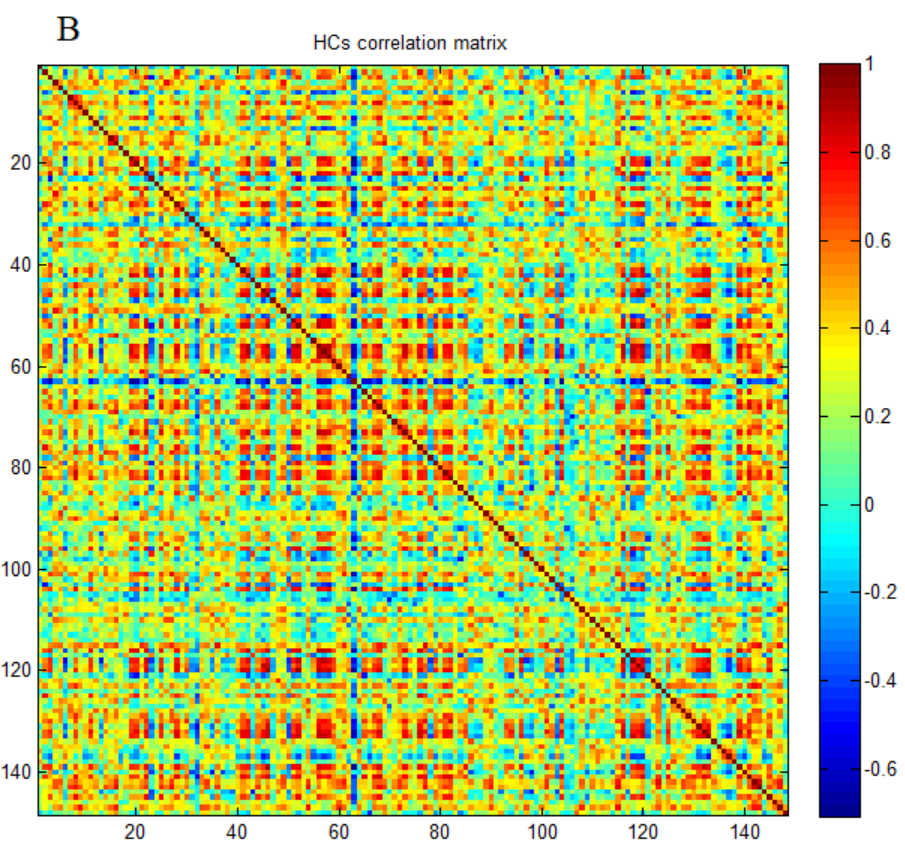


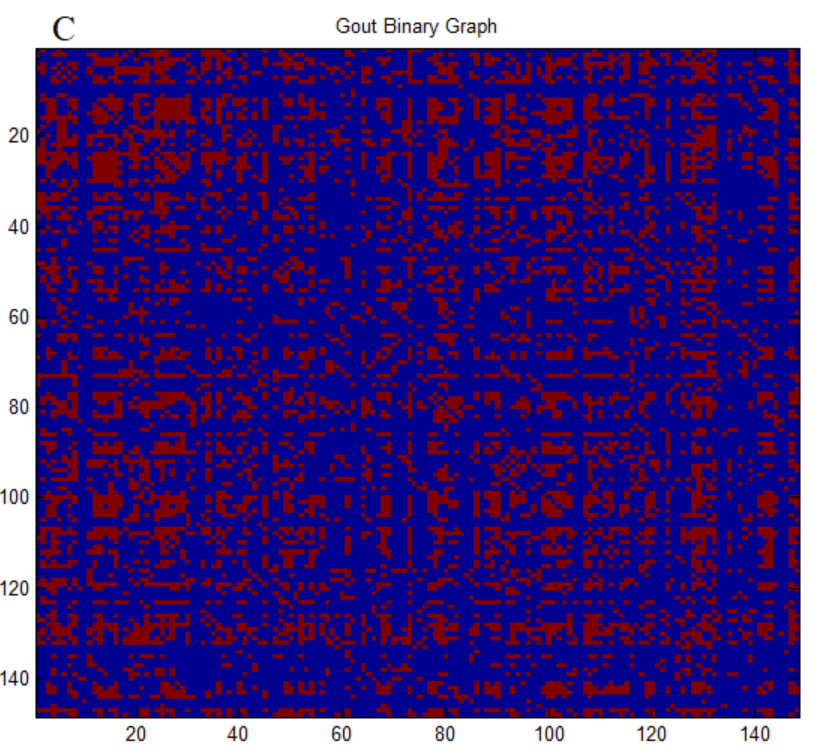

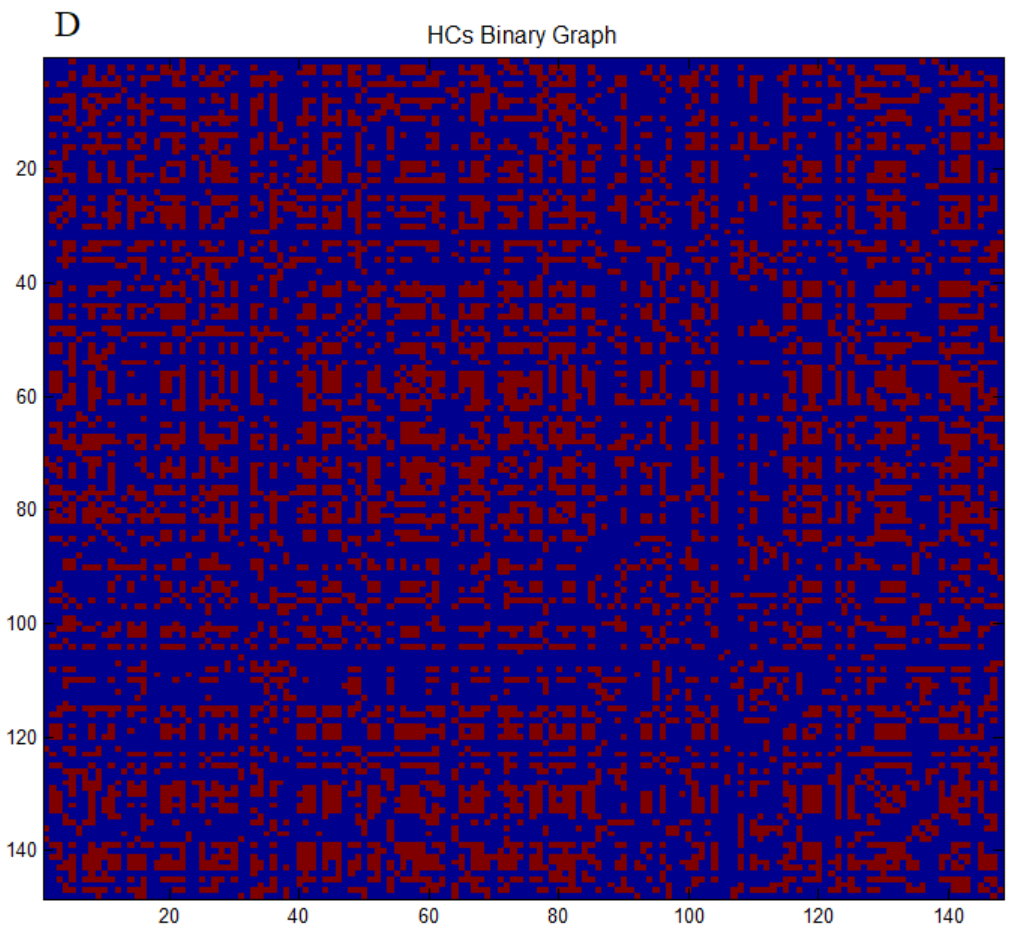


**Supplementary material. 2.** Correlation and binary matrices for gout patients and health controls (HCs). Correlation matrices for Gout (A) and HCs (B), and binary adjacency matrices thresholded at Dmin (0.1) for Gout (C) and HCs (D). The color bar denotes the correlation coefficient and represents the strength of the connections.

| 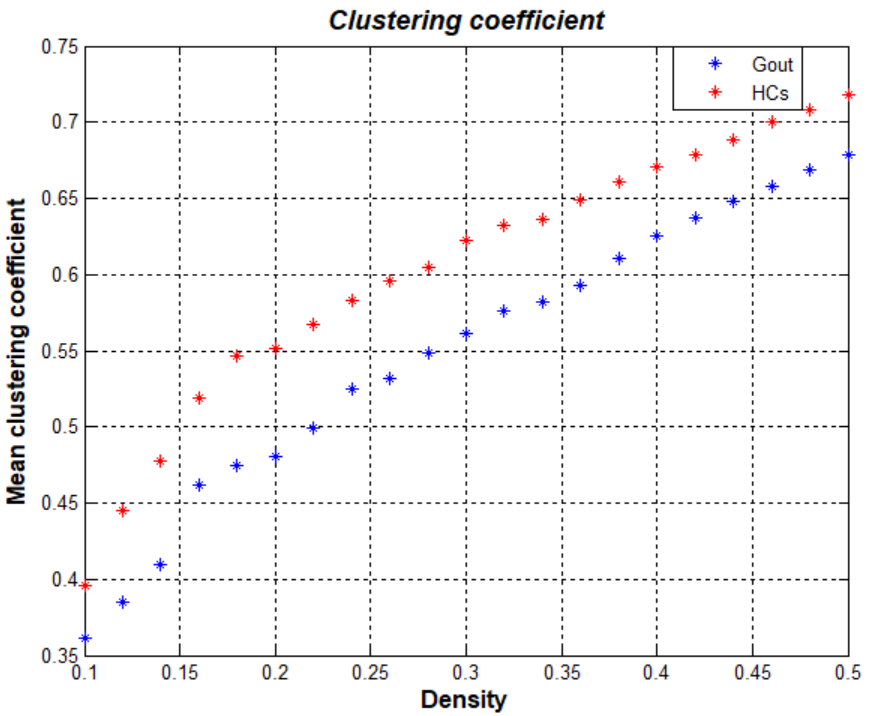  A | 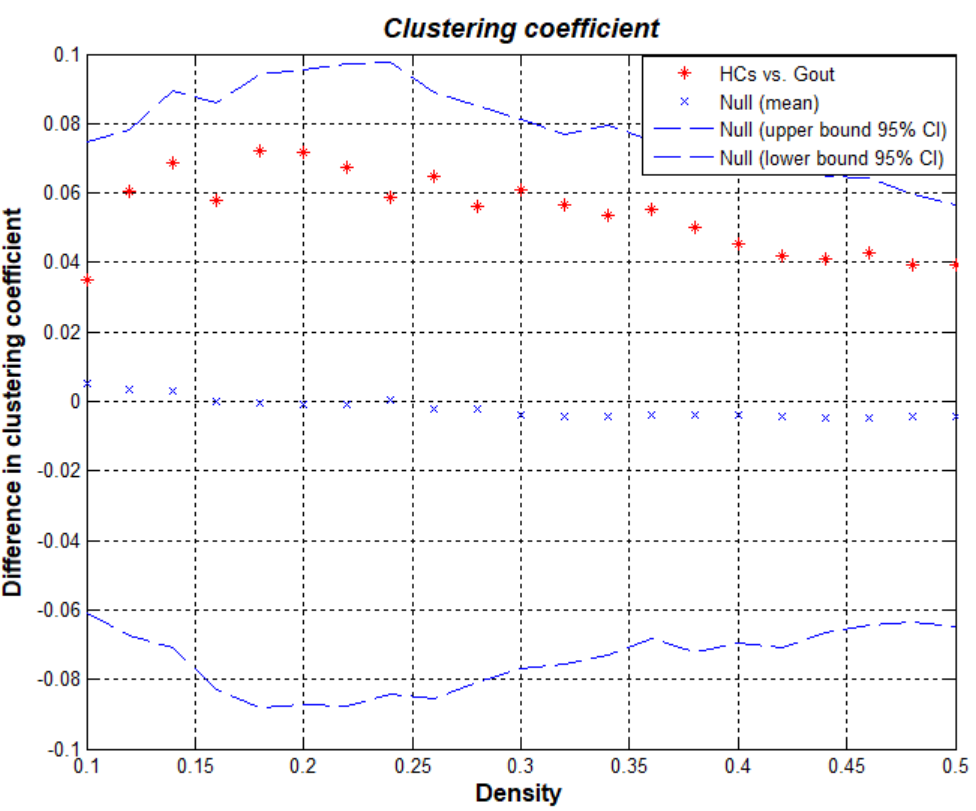  B |
| --- | --- |
| 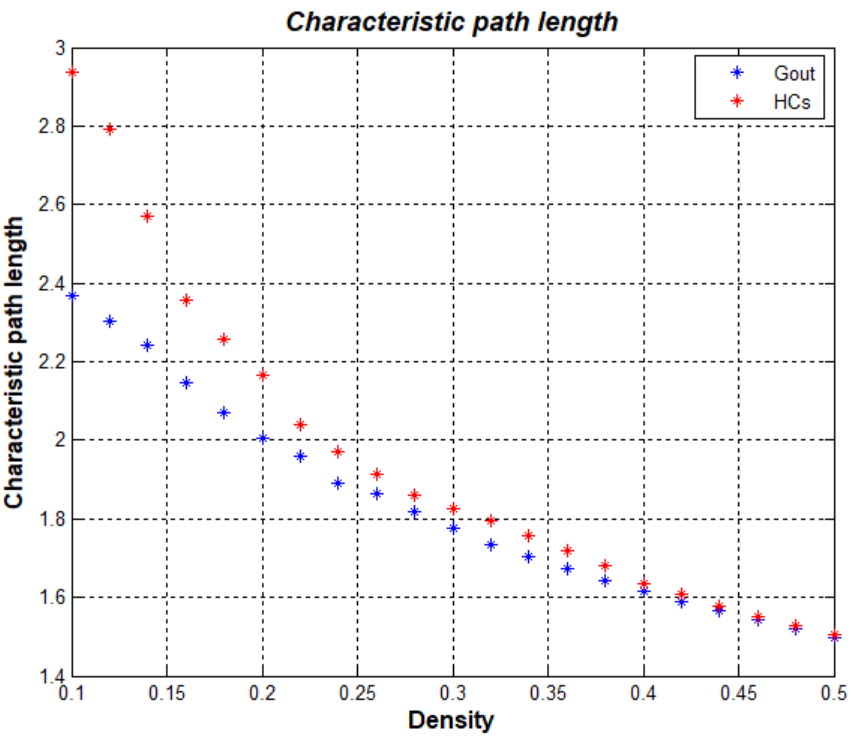  C | 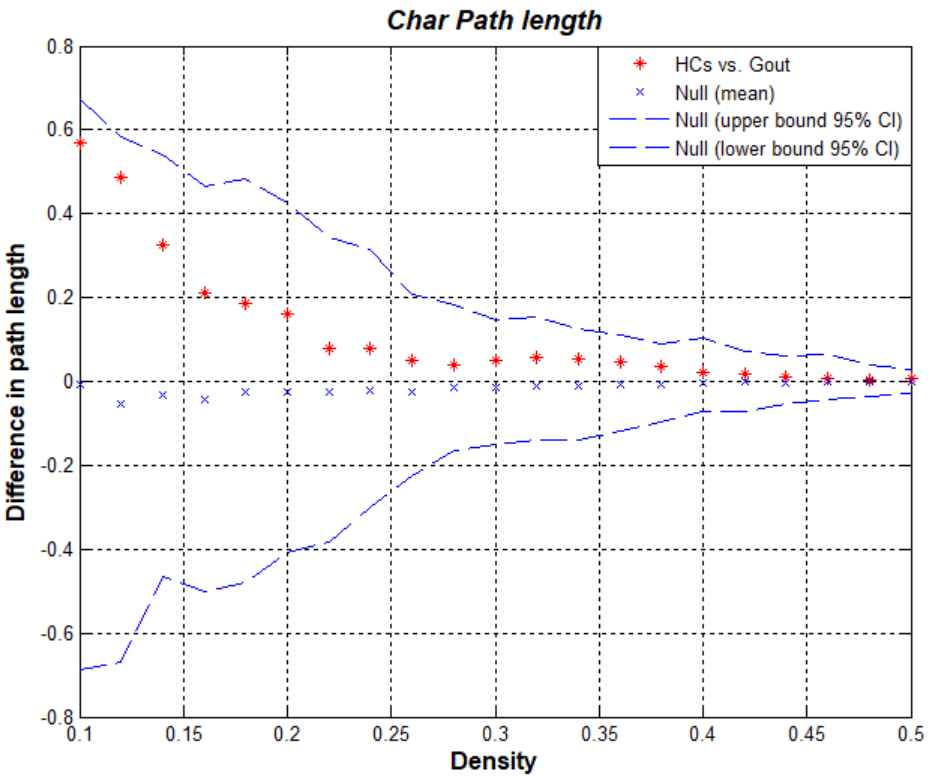  D |
| 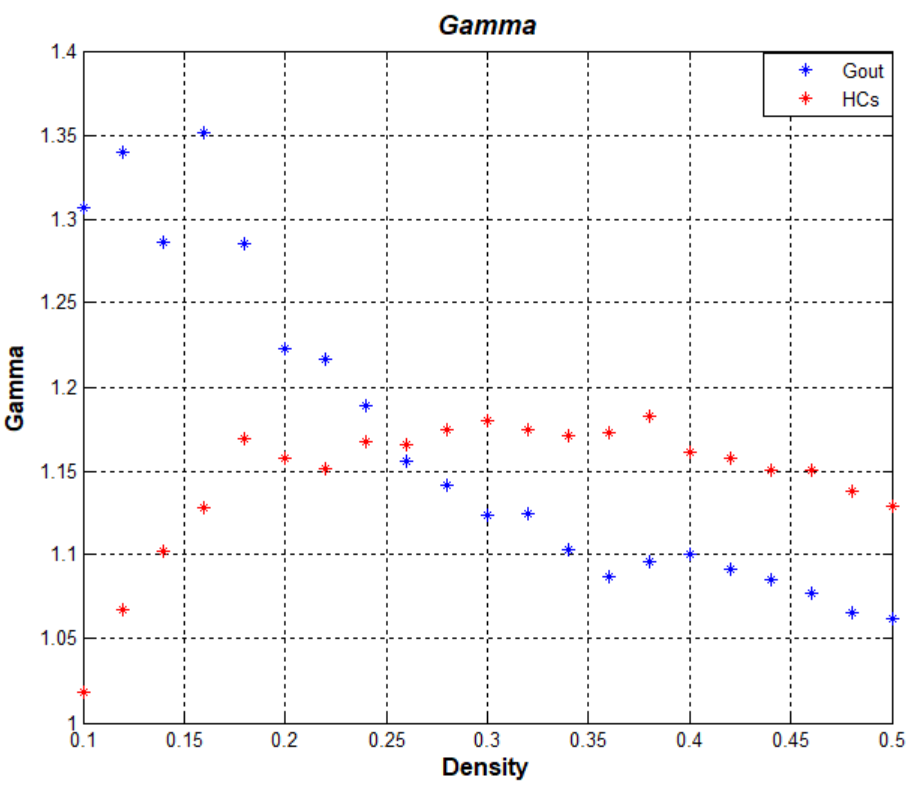  E | 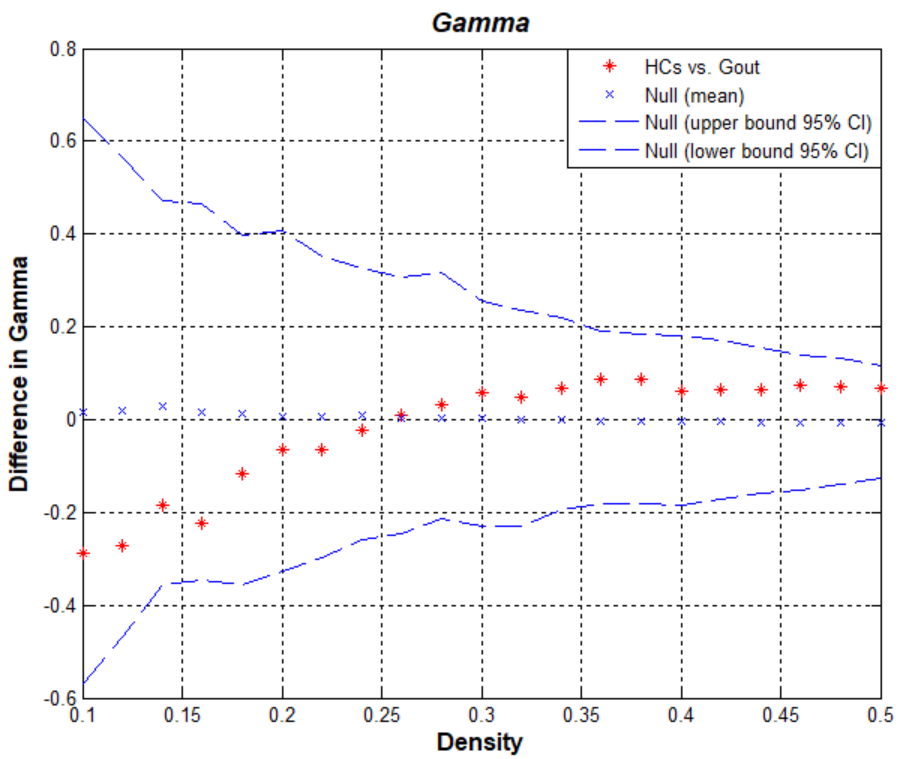  F |
| 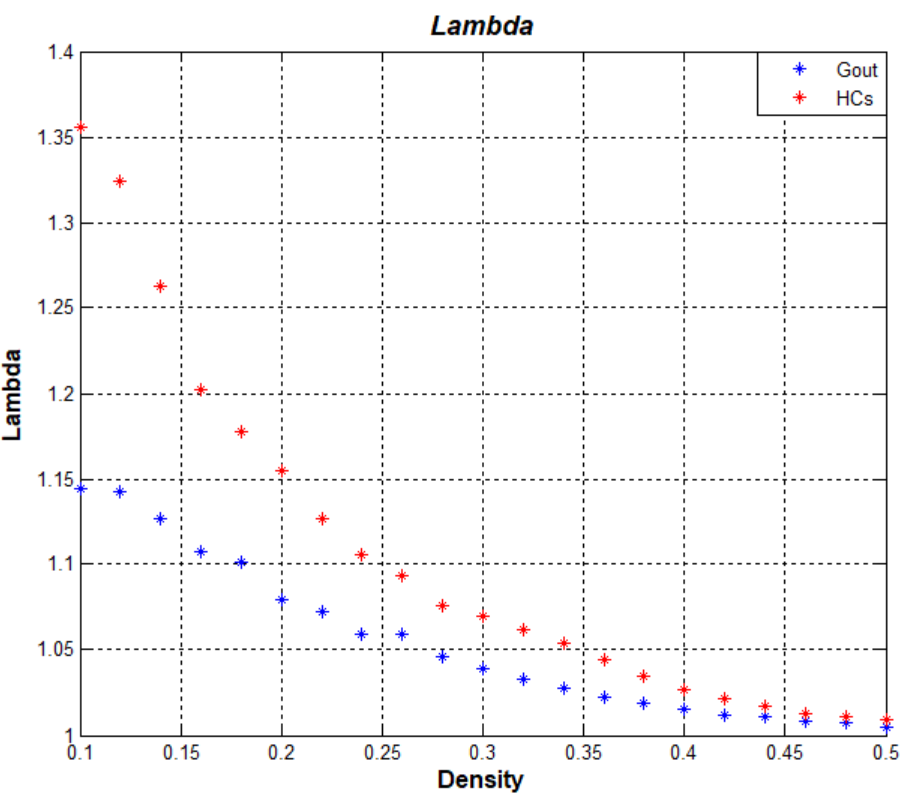  G | 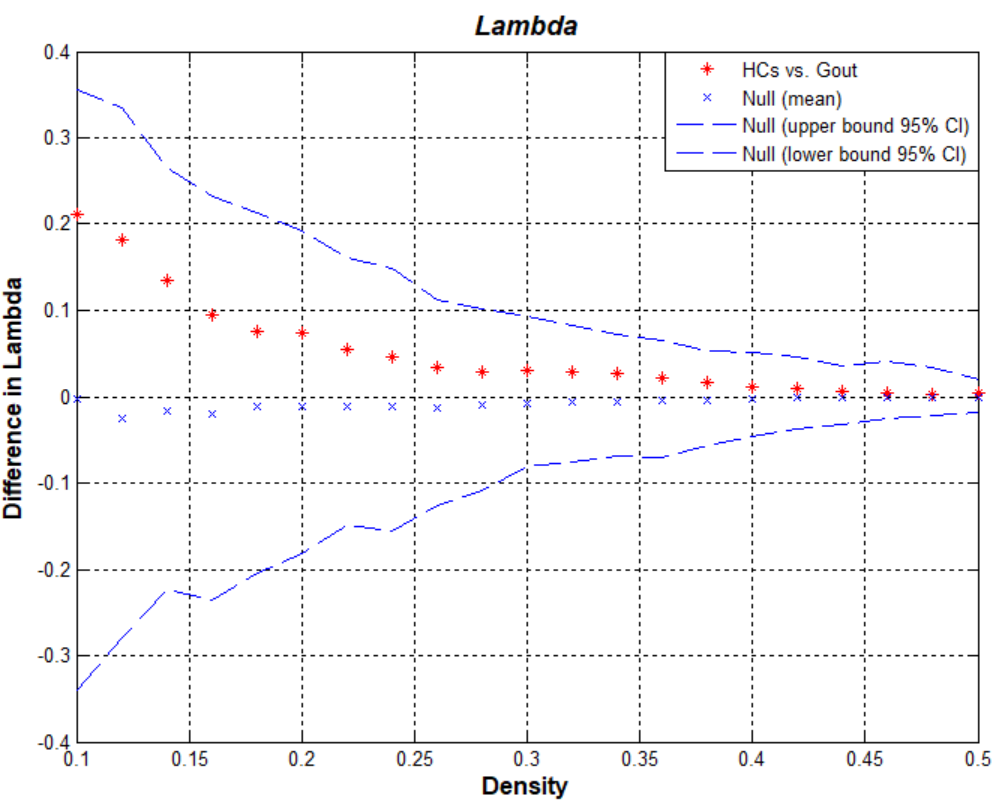  H |
| 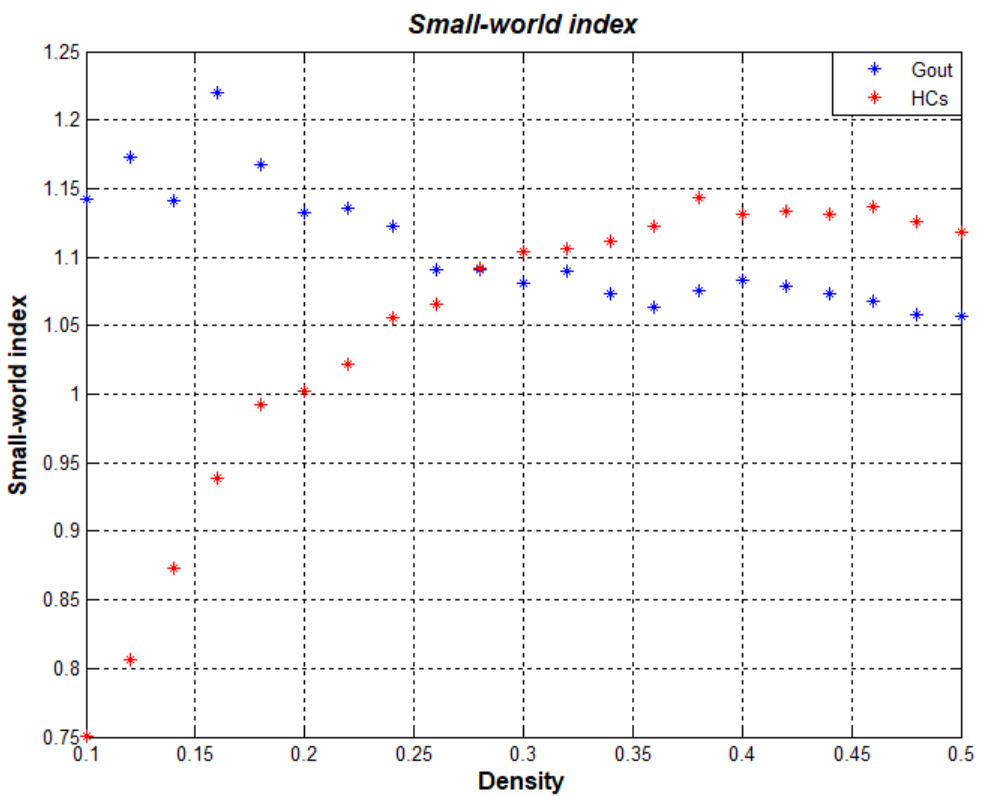  I | 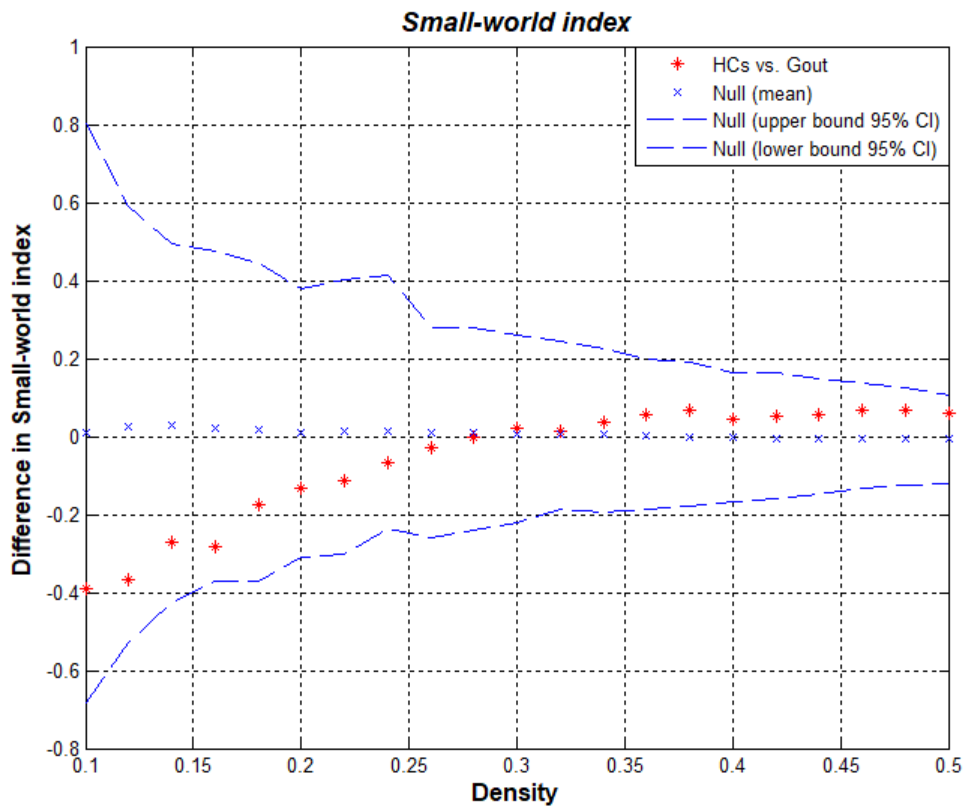  G |
| 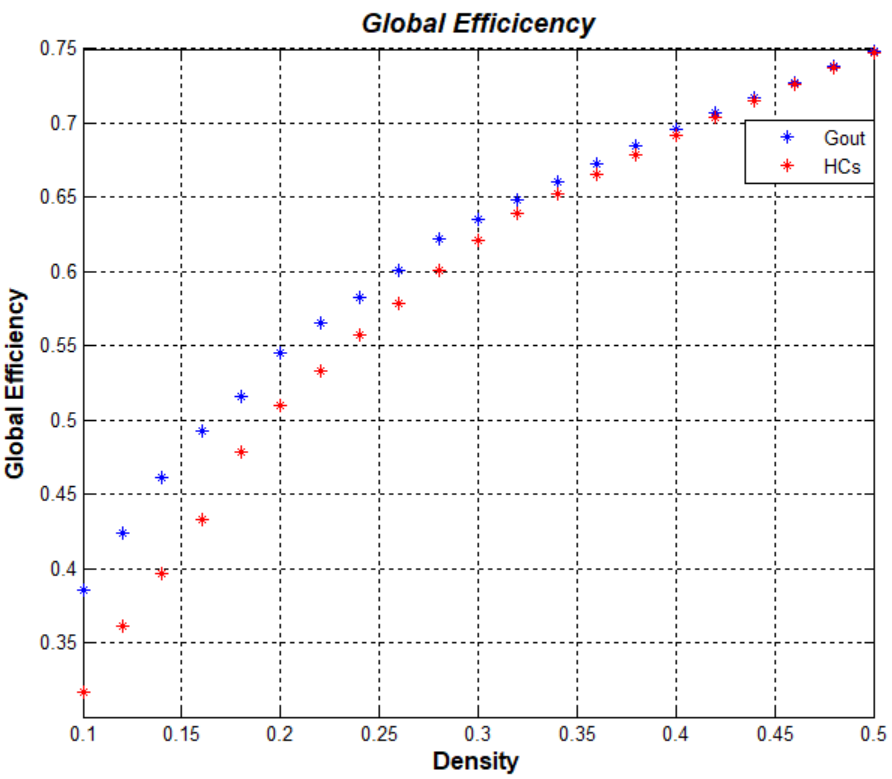  K | 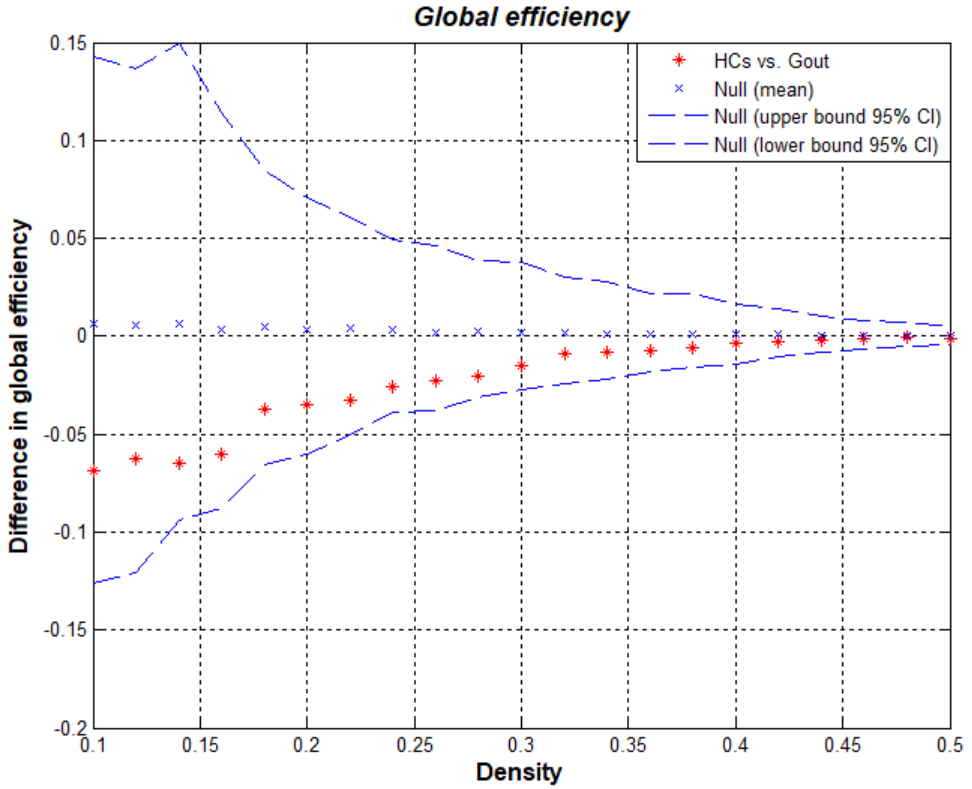  L |
| 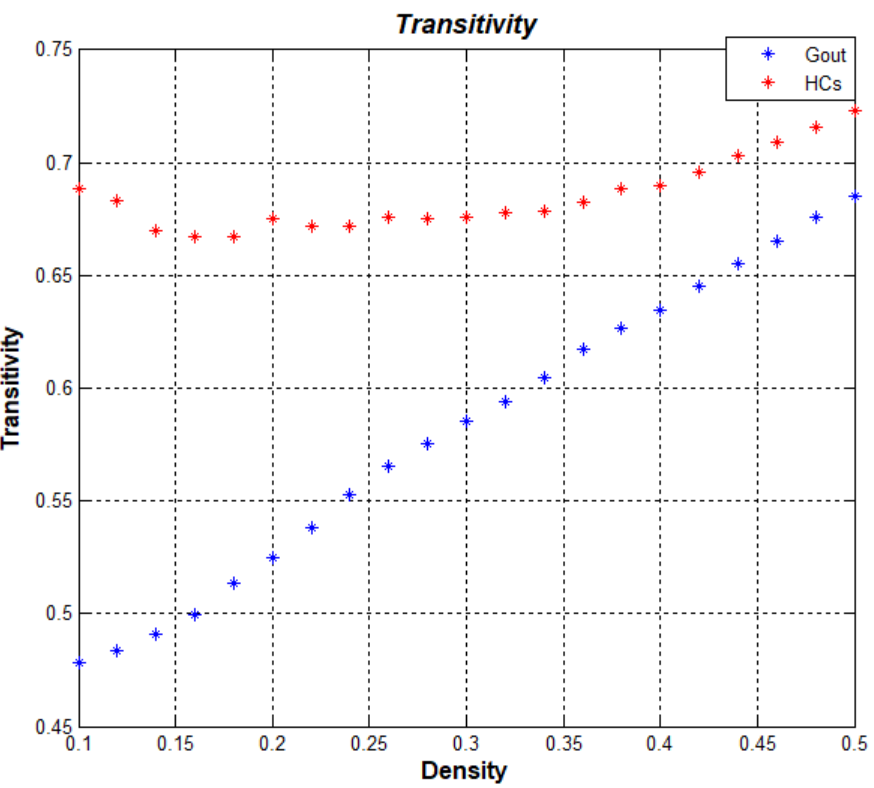  M | 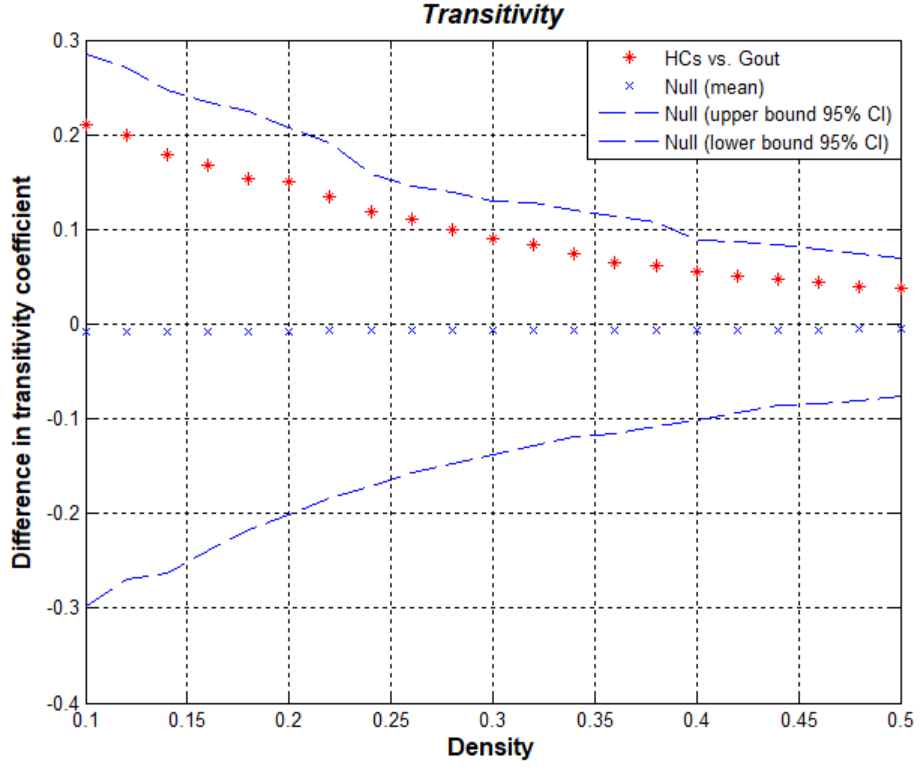  N |
| 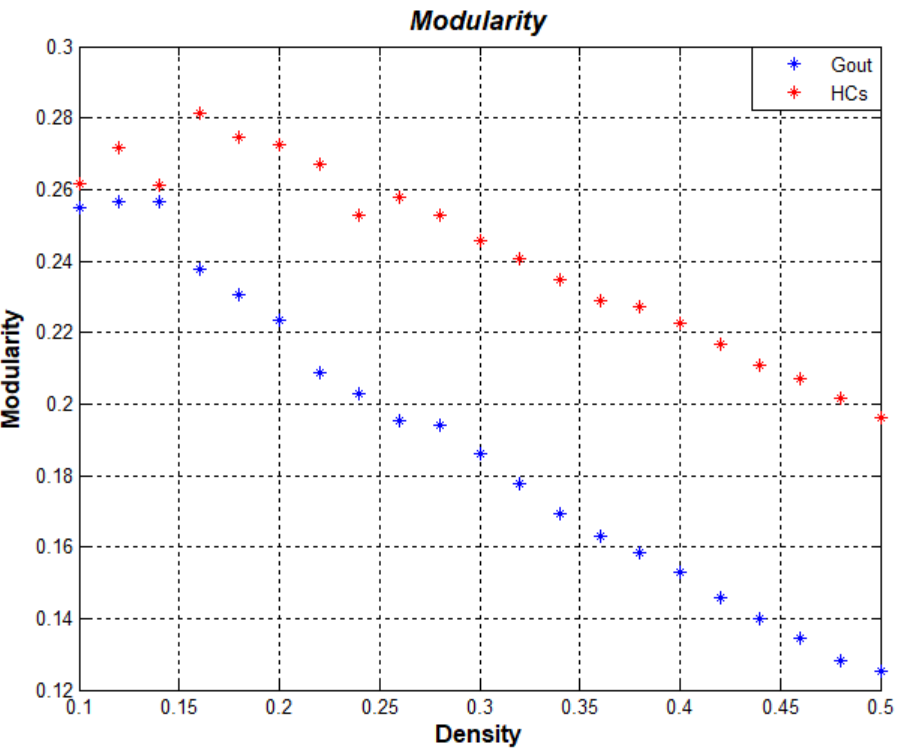  O | 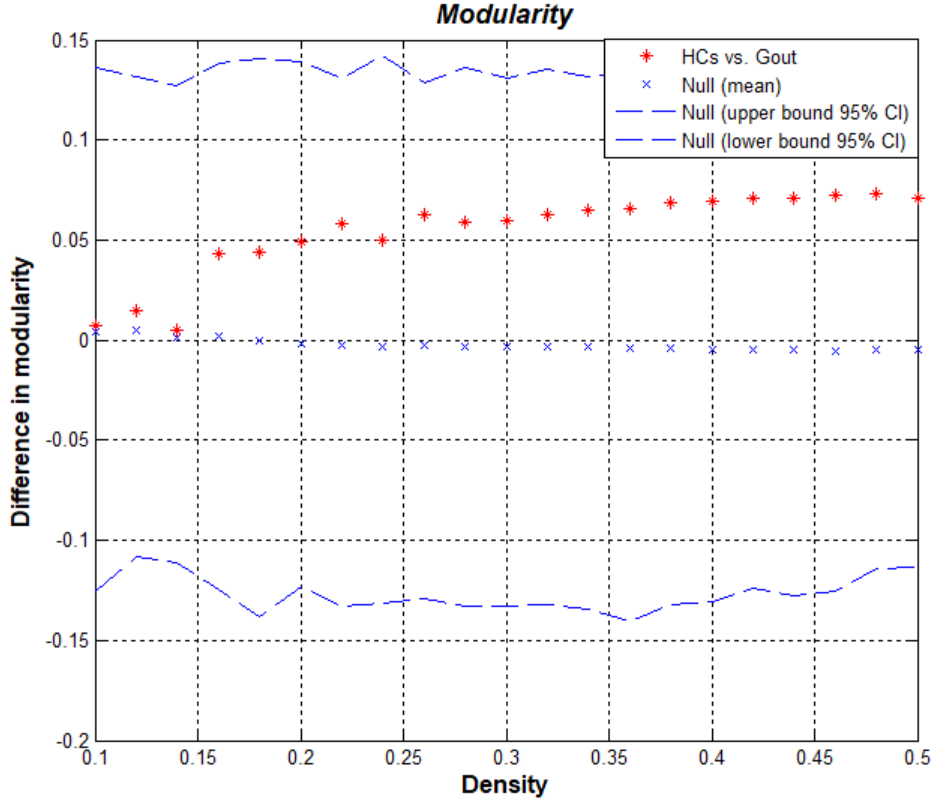  P |
| **Supplementary material. 3.** Global network measures of two groups, and between-group differences in these measures. Clustering coefficient (A, B), Characteristic path length (C, D), Gamma (E, F), Lambda (G, H), Small-world index (I, J), Global efficiency (K, L), Transitivity (M, N) and Modularity (O, P) of the BCP and HC networks. The red * lying outside of the confidence intervals indicates the difference between the two groups in this density is significant (B,D,F,H,G,L,N,P). All above measures were not significantly different between the two groups. Gout, gout group; HCs, healthy controls.  The results using the Desterieux Atlas were roughly the same as those using the Desikan-Kiliany Atlas template, except that using the Desterieux Atlas failed to find a significant difference in global efficiency between the two groups. | |

| 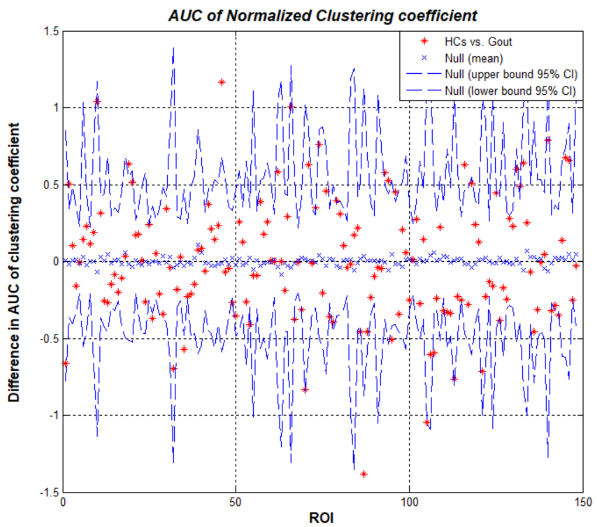  A | 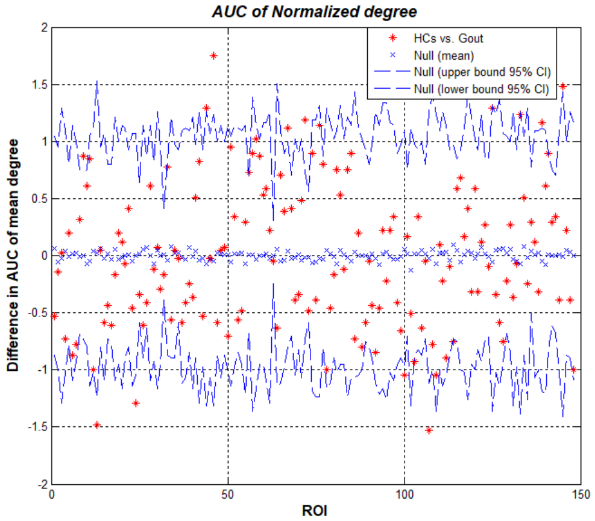  B |
| --- | --- |
| 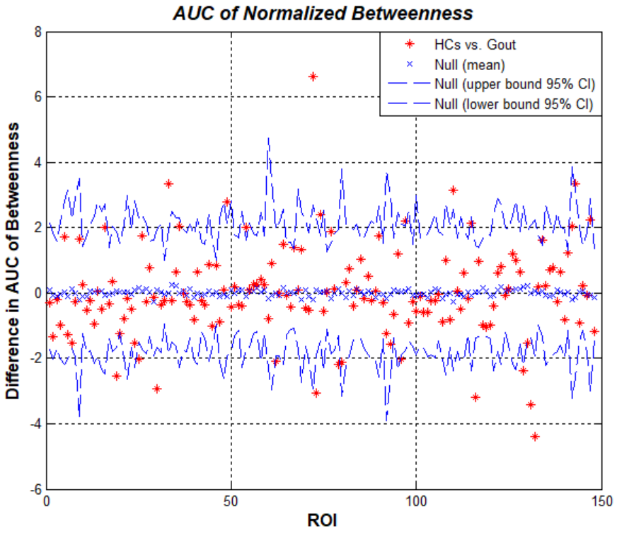  C |  |

**Supplementary material. 4.** Between-group differences of measures across a range of network densities: normalized clustering coefficient (A), degree (B), and betweenness (C). The red * lying outside of the confidence intervals indicate regions which the difference between the two groups in this density is significant. All regions survive following FDR correction (p < 0.05). Gout, gout group; HCs, healthy controls. AUC, area under the curve.

Due to the finer partitions and more brain regions, more brain regions with significant differences in regional network measures were obtained by using Desterieux Atlas compared with using Desikan-Kiliany Atlas, but the distribution of abnormal brain regions was basically consistent. For example, using both templates, the reduction in Clustering coefficient in patients with gout compared with HCs was mainly in the occipital lobe, while most of the increased regions were located in the temporal and frontal lobes.

Supplementary material. 5. Between-group differences in regional network measures

|  | Gout ＜ HCs | Gout ＞ HCs | P-value |
| --- | --- | --- | --- |
| Clustering coefficient | lh_G_occipital_middle  lh_S_cingul-Marginalis  rh_Pole_occipital | lh_G_pariet_inf-Angular  lh_G_temp_sup-Plan_pola  lh_S_front_sup  lh_S_postcentral  rh_G&S_paracentral  rh_G_front_inf-Orbital  rh_G_oc-temp_med-Lingual  rh_G_temp_sup-G_T_transv  rh_G_temp_sup-Plan_tempo | 0.05 |
| Degree | lh_G_cingul-Post-dorsal  lh_S_calcarine  lh_S_cingul-Marginalis  lh_S_temporal_inf | lh_G_front_inf-Orbital  lh_G_orbital  rh_G_temp_sup-G_T_transv | 0.05 |
| Betweenness | lh_G_temp_sup-G_T_transv  lh_S_circular_insula_sup  lh_S_front_sup  lh_S_temporal_inf  lh_S_temporal_transverse  rh_G&S_paracentral  rh_G_temp_sup-Plan_tempo  rh_S_precentral-sup-part | lh_G_occipital_middle  lh_G_pariet_inf-Angular  lh_G_precuneus  lh_S_temporal_sup  rh_G&S_transv_frontopol  rh_Pole_occipital  rh_S_oc_middle&Lunatus  rh_S_oc_sup&transversal | 0.05 |

Gout, gout group; HCs, healthy controls; lh, left hemisphere; rh, right hemisphere.

**Supplementary material. 6.** Network hubs in Desikan-Kiliany Atlas and Desterieux Atlas

| Network hubs | | |
| --- | --- | --- |
|  | Gout group hubs | HCs group hubs |
| Desikan-Kiliany Atlas | left parahippocampal  left insula  right superiorparietal | left superiorfrontal  right superiorfrontal  right supramarginal |
| Desterieux Atlas | lh_G_precuneus  lh_S_temporal_sup  rh_G&S_transv_frontopol  rh_G&S_cingul-Ant  rh_G_parietal_sup  rh_S_intrapariet&P_trans  rh_S_oc_middle&Lunatus | lh_G_temp_sup-G_T_transv  lh_G_temp_sup-Plan_tempo  lh_S_circular_insula_sup  lh_S_temporal_inf  rh_S_front_sup  rh_S_precentral-inf-part  rh_S_precentral-sup-part  rh_S_temporal_sup |

The number of network nodes obtained using the two templates is different, and the positioning only partially overlaps (right superiorparietal in the gout group and right frontal in the HCs group), but the results show that the network nodes of gout patients are different from HCs.
